# Data-driven estimation of core body temperature during physical activity under heat exposure: A systematic review and standardized evaluation

**DOI:** 10.1016/j.buildenv.2026.114591

**Published:** 2026-06-01

**Authors:** Yuanzhe Zhao, Weihao Li, Jeroen HM Bergmann

**Affiliations:** a Department of Engineering Science, University of Oxford, Oxford, UK; b School of Automation Science and Electrical Engineering, Beihang University, Beijing, China; c Department of Technology & Innovation, University of Southern Denmark, Odense, Denmark

**Keywords:** Core body temperature, Skin temperature, Heart rate, Heat stress, Hot environment, Machine learning

## Abstract

Accurate, real-time estimation of core body temperature (CBT) during physical activity is essential for monitoring heat strain and mitigating the risk of heat-related illness under hot environmental conditions. Although numerous data-driven algorithms using wearable sensors have been proposed, their practical reliability remains unclear due to substantial methodological heterogeneity and the absence of standardized evaluation.

This study combined a systematic review with a standardized quantitative benchmark. A total of 38 studies employing non-invasive inputs for CBT estimation were identified. Of these, 14 eligible models, including Kalman filter–based methods, statistical models, and machine-learning approaches, were re-implemented and evaluated under identical preprocessing and evaluation settings using two independent datasets: Dataset 1 (treadmill walking, n=16) and Dataset 2 (cycling, n=13).

The benchmark revealed notable differences between originally reported performance and reproduced performance under standardized conditions. For the widely used heart-rate–based extended Kalman filter, the root mean square error (RMSE) increased from typically reported values of ∼0.21–0.41 ${\;}^{\circ }$C to 0.41 ${\;}^{\circ }$C on Dataset 1 and 0.66 ${\;}^{\circ }$C on Dataset 2. Incorporating skin temperature improved tracking accuracy in some configurations, but performance gains were highly dependent on measurement site and dataset. Sensitivity for detecting elevated CBT (≥38.0 ${\;}^{\circ }$C) varied markedly across methods, particularly for the cycling protocol.

In conclusion, no single CBT estimation approach consistently outperformed others across all settings. Heart-rate–only models provided a stable baseline under limited sensing conditions, whereas multimodal approaches offered conditional benefits in more controlled scenarios. This work establishes a standardized benchmark framework to support fair comparison, method selection, and future development of (wearable) CBT estimation technologies.

## Highlights

• Systematic review of core temperature estimation during physical activity in hot environments.
• Standardized benchmark across two controlled heat-exposure datasets reveals strong dataset dependence.
• Results inform the deployment of wearable heat-strain monitoring in occupational and built-environment settings.

## 1 Introduction

Human exposure to hot environments during sustained physical activity poses substantial risks to health, safety, and productivity across occupational, military, and other real-world settings [1], [2], [3]. Exertional heat illness (EHI), particularly exertional heat stroke (EHS), represents a critical outcome of excessive internal heat strain and remains a significant cause of heat-related morbidity and mortality during strenuous activity under high environmental heat exposure [4], [5]. EHS is characterized by a marked elevation in core body temperature, typically exceeding 40 ${\;}^{\circ }$C, accompanied by signs of central nervous system dysfunction due to hyperthermia [6], [7]. The risk of morbidity and mortality increases sharply with the duration for which body temperature remains above critical thresholds, particularly beyond 41 ${\;}^{\circ }$C, but can be substantially reduced through rapid and effective cooling [8], [9]. Accordingly, contemporary occupational hygiene and environmental physiology emphasize the importance of continuous heat-strain surveillance to guide work–rest scheduling, environmental controls, and individualized safety interventions in hot environments [10], [11], [12].

To support heat-strain surveillance, practitioners often begin by assessing environmental conditions. The most widely used metric is the Wet-Bulb Globe Temperature (WBGT), which integrates air temperature, humidity, solar radiation, and wind speed into a single index of external heat load [13], [14]. WBGT helps guide work–rest scheduling and recovery strategies by identifying environmental conditions under which excessive heat strain is likely to develop [15], [16]. However, WBGT reflects only environmental heat stress and cannot account for key determinants of internal heat strain, such as metabolic rate, activity intensity, heat-acclimation status, individual tolerance, or clothing and equipment burden. It also fails to capture rapid within-session fluctuations in thermal load or indicate when an individual is approaching a dangerous rise in core body temperature [17], [18]. As a result, heat-related illnesses may still occur even under WBGT-guided management and extensive preventive measures, as observed in large-scale events such as the Tokyo 2020 Olympic and Paralympic Games [19], [20]. Thus, while WBGT is valuable for environmental risk classification, it cannot replace physiologically grounded monitoring capable of detecting changes in internal temperature in real time.

In addition to environmental indices, heat-strain management in occupational settings commonly incorporates physiological safety principles aimed at maintaining thermal equilibrium during prolonged activity. In practice, work–rest scheduling and exposure limits are often defined to prevent excessive internal heat storage and to avoid sustained elevations in core body temperature beyond approximately 38 ${\;}^{\circ }$C over extended periods [21], [22], [23]. A widely adopted example is the Predicted Heat Strain (PHS) model, which estimates the evolution of core body temperature and sweat loss from environmental conditions (including air temperature, humidity, air velocity, and radiant heat exposure), together with individual physiological characteristics such as metabolic rate and clothing insulation, to support the prediction of allowable exposure durations in occupational environments [24], [25]. Unlike environmental indices such as WBGT, which reflect only external thermal load, the PHS framework explicitly accounts for internal heat storage and thermoregulatory strain. However, PHS is primarily intended for task-level exposure assessment rather than individualized continuous monitoring during dynamic physical activity. Consequently, accurate and real-time assessment of CBT remains essential for effective heat-strain mitigation and the prevention of exertional heat illness.

In practice, however, monitoring core temperature remains challenging. Traditional thermometry measurements such as oral, axillary, and tympanic, are convenient in clinical or resting conditions but do not reliably track CBT during dynamic physical activity due to airflow, sweat evaporation, motion artefact, and variable peripheral blood flow [26], [27], [28]. Their agreement with true CBT deteriorates particularly during exercise-induced thermal transients, making them unsuitable for real-time heat-strain management [29], [30], [31]. For this reason, rectal and gastrointestinal temperatures are widely regarded as the laboratory gold standards for CBT assessment, and the temperature obtained from ingestible telemetry pills is commonly used in field-based studies [7], [32]. Yet all of these gold-standard approaches are invasive, burdensome, and unsuitable for many environments: rectal probes are uncomfortable and impractical during movement; ingestible pills require repeated dosing, have variable transit times, and rely on dedicated receivers [26], [33]. Even promising engineering alternatives such as zero-heat-flux (ZHF) thermometry require specialized heated sensors, rigid fixation, and consistent thermal contact [34], [35]. These conditions are difficult to maintain during vigorous movement, heavy sweating, or prolonged outdoor physical activities. Thus, although these methods offer high accuracy, their reliance on specialized hardware and restrictive usage conditions limits their feasibility for routine monitoring.

Beyond direct measurement approaches, a parallel line of research has explored fully mechanistic thermophysiological models that predict CBT from fundamental principles of heat production, storage, and transfer [36], [37], [38]. These models typically represent the body as a system of one or more thermal compartments and express metabolic heat generation, conductive and convective heat exchange, blood flow–mediated heat transport, and evaporative cooling through coupled differential equations. However, these models require numerous physiological parameters such as metabolic rate, perfusion rate, and workload that are difficult to measure or estimate in real time, and the high computational demands of some finite-element models make them impractical for deployment on wearable devices [39], [40]. Consequently, despite their strong physiological grounding, purely mechanistic models have seen limited application in real-world operational monitoring, where low-latency and individualized estimates of CBT are required.

Over the past decades, substantial research has sought to bridge this monitoring gap through data-driven algorithms capable of inferring CBT from wearable sensor data and environmental inputs. Several studies relied on statistical modeling incorporating multi-inputs, such as skin temperature at varied locations, heat flux, and environmental conditions [41], [42], [43], [44]. These models provided valuable empirical insights into human thermoregulation; however, their static functional forms inherently limited their ability to capture the dynamics arising from changes in workload or environmental conditions and restricted their generalizability across individuals. Moreover, these models frequently relied on multiple peripheral inputs, which further constrained their feasibility for real-world wearable deployment. Subsequent applications of state-space modeling and Kalman filtering enabled the fusion of sequential heart-rate histories, skin temperatures, and individualized priors into adaptive estimators [45], [46], [47], [48]. Seminal work by Buller and colleagues demonstrated that heart-rate driven models could maintain tracking accuracy within approximately 0.2–0.3 ${\;}^{\circ }$C root mean square error (RMSE) during real-world walking, agricultural work, and military ruck marches, inspiring interest in embedding such algorithms into wearable devices and real-time command platforms [49], [50], [51].

The methodological landscape has since diversified substantially. Existing non-invasive CBT estimation approaches broadly span statistical regression models, Kalman filter or extended Kalman filter models, machine learning methods, and hybrid formulations that integrate biophysical structure with data-driven components [52], [53], [54], [55]. Reported performance below 0.2 ${\;}^{\circ }$C RMSE is increasingly common. However, these results largely stem from carefully controlled laboratory or heat chamber experiments, where ground-truth CBT is reliably captured using ingestible pills or rectal probes and environmental conditions are precisely controlled. Despite promising progress, critical challenges remain; algorithm transparency varies widely, independent validation cohorts are scarce, and comparisons across model classes are hindered by inconsistent reporting of input types, sampling rates and sensor placements. Without standardized evaluation frameworks, practitioners cannot fairly benchmark models, replicate findings, or integrate algorithms into monitoring systems and wearable platforms.

Two prior reviews have attempted to summarize this evolving field. Falcone et al. focused on occupational CBT prediction and highlighted the absence of uniform, model-to-model comparisons, particularly under protective clothing or heat-stress conditions [56]. Dolson et al. surveyed wearable technologies for CBT estimation in athletic and clinical applications, underscoring the promise of data-driven monitoring but noting substantial methodological heterogeneity and limited external validation [57]. Since their publication, further work—including refinements of regression-based and Kalman filter approaches as well as emerging machine-learning and deep-learning models—has broadened the available evidence. These developments underscore the need for an updated synthesis focused on the application in physical activities under heat exposure.

To address these gaps, the present work focuses specifically on data-driven estimation of core body temperature during physical activity under heat stress and combines a comprehensive systematic review with a standardized evaluation framework. The systematic review synthesizes how wearable-derived physiological signals and environmental measurements have been exploited for CBT estimation, and systematically summarizes the reported algorithmic methods, input signal configurations, and model performance across occupational, military, and athletic applications. The standardized evaluation benchmarks eligible data-driven algorithms using two previously published datasets involving structured treadmill walking and staged cycling protocols, with rectal temperature as the reference CBT and heart rate and multi-site skin temperatures as the non-invasive inputs [58], [59], [60]. Together, these components provide the first integrated synthesis and quantitative comparison of data-driven CBT estimation methods, offering a unified reference for future algorithm development, validation, and deployment in real-world heat-strain monitoring.

## 2 Methods

### 2.1 Systematic review

#### 2.1.1 Review framework

This systematic review was conducted in accordance with the Preferred Reporting Items for Systematic Reviews and Meta-Analyses (PRISMA) guidelines [61]. The review aimed to identify real-time data-driven methods for estimating core body temperature (CBT) during exercise, occupational heat stress, military tasks, or other dynamic activities using physiological signals obtainable from wearable or non-invasive sensors.

The objectives were to (1) characterize the modeling paradigms used in CBT estimation, (2) summarize the physiological and environmental inputs across studies, as well as the ground-truth CBT measurement methods, (3) synthesize and compare the validation metrics reported across studies, and (4) identify methods suitable for reproducible implementation in the standardized benchmark analysis.

#### 2.1.2 Search strategy

A structured literature search was conducted in *Web of Science*, *PubMed*, and *IEEE Xplore* from database inception to June 2025. The following Boolean query was applied systematically across all databases, with database-specific syntax adapted as needed:

*Human AND (“core body temperature” OR “deep body temperature” OR “core temperature” OR “rectal temperature”) AND (estimation OR prediction OR model)*

All retrieved records were imported into EndNote for reference management and deduplication. In addition, the reference lists of included articles and relevant review papers were manually screened to identify further eligible studies.

#### 2.1.3 Eligibility criteria

Eligibility criteria were defined to identify studies relevant to real-time, data-driven estimation of CBT during physical activity. Studies were eligible if they:

1. developed or evaluated a method for predicting or estimating human CBT with sufficient methodological detail for interpretation;
2. employed a data-driven modeling approach (e.g., statistical models, Kalman or extended Kalman filtering, machine learning, deep learning, or hybrid methods);
3. used physiological or environmental inputs obtainable in real time from wearable or otherwise non-invasive sensors (e.g., heart rate, skin temperature, heat flux);
4. collected data during dynamic physical activity (e.g., exercise, occupational work, military tasks);
5. used a valid reference CBT measurement (rectal, esophageal, or gastrointestinal telemetry pill);
6. and were published as peer-reviewed journal articles or full conference papers in English.

#### 2.1.4 Study screening

**Fig. 1:**
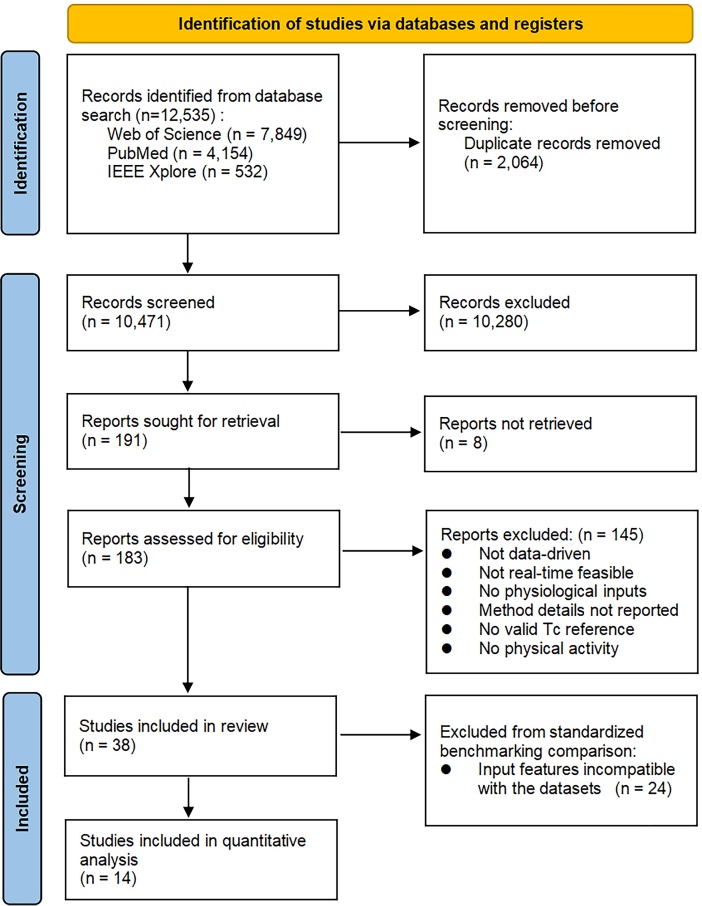
PRISMA flow diagram illustrating the identification, screening, eligibility assessment, and inclusion of studies in the systematic review.

Screening followed the PRISMA workflow. Titles and abstracts were independently screened by Zhao and Li to remove duplicates, irrelevant records, and non-data-driven studies. Full text articles were then assessed in detail against all eligibility criteria. Any disagreements were resolved through discussion, and when necessary, adjudicated by Bergmann. The PRISMA flow diagram summarizing identification, screening, exclusion, and inclusion is shown in Fig. 1.

#### 2.1.5 Data extraction

A structured extraction form was used to capture key methodological and experimental information from each included study. Extracted data included participant and experimental characteristics such as participant type (e.g., athletes, military personnel, occupational workers), sample size, sex distribution, age, body mass, and height. Environmental conditions (ambient temperature and relative humidity) and clothing or protective equipment were recorded when available. Exercise protocols were documented in detail, including the type of activity (e.g., walking, cycling), prescribed intensity (e.g., speed, workload, target heart rate), duration, and whether efforts were continuous or intermittent with rest or recovery periods. The duration of continuous monitoring was also extracted.

Information on measurement devices included the specific wearable or non-invasive sensors used (e.g., heart rate, skin temperature, heat flux), the reference CBT method (rectal, esophageal, or gastrointestinal telemetry pill), and the sampling rate of physiological measurements. Model-related information comprised the modeling approach (e.g., statistical models, Kalman or extended Kalman filtering, machine learning, deep learning, or hybrid methods), input variables, and implementation details when provided.

Extracted performance metrics included the root mean square error (RMSE), mean absolute error (MAE), Bland–Altman mean difference (MD) and standard deviation (SD), and the coefficient of determination ($R^{2}$). Many included studies evaluated multiple modeling approaches or reported several variants of the same model (e.g., identical algorithmic architecture evaluated with different input configurations). To ensure consistent comparison across publications and to avoid overweighting studies that tested a large number of model variants, a predefined extraction rule was applied: (1) For each modeling category within a study (e.g., EKF/KF, statistical, machine learning, hybrid), only the primary or best-performing model variant was extracted for inclusion in the summary tables and comparative analyses. (2) When a study reported multiple input configurations for the same primary model (e.g., heart rate only; heart rate and three skin-temperature sites; heart rate and full multi-site skin temperature), all reported configurations and their associated performance were recorded. These details were retained because input composition substantially influences data requirements and practical deployability. This extraction strategy ensures fairness across studies, avoids inflating the influence of publications that evaluated numerous model permutations, and maintains transparency regarding the relationship between model performance and input availability.

A fully expanded table containing all extracted study characteristics (including participant profiles, experimental protocols, sensor configurations, input variables, and reported performance metrics) is provided in the supplementary materials.

### 2.2 Standardized benchmark

To enable fair, head-to-head comparison of data-driven core body temperature (CBT) estimation models identified in the systematic review, a standardized benchmark framework was implemented using two controlled experimental datasets.

#### 2.2.1 Datasets

Dataset 1 comprises treadmill-based heat-exposure trials conducted under six controlled environmental settings [58], [59]. The settings varied in ambient temperature (25–40${\;}^{\circ }$C), relative humidity (20–50%), solar radiation (0 or 530 W⋅m^−2^), and clothing type (permeable or impermeable). In addition to heart rate and rectal temperature, the dataset includes a broad range of physiological measurements. These include multi-site skin temperatures recorded at eleven anatomical locations (abdomen, calf, chest, foot, hand, head, lower arm, lower back, thigh, upper arm, and upper back). Several experiment-specific variables such as insulated skin temperatures, insulated climatic temperatures, environmental conditions and breathing rate were also collected in the original study but were not used in the benchmark analysis. Each trial consisted of alternating rest and treadmill walking phases, with cooling periods introduced until rectal temperature decreased by 0.4${\;}^{\circ }$C before subsequent exercise bouts.

Trials with missing data in any variable required for the benchmark analysis were excluded from the dataset. After applying these completeness criteria, the six environmental settings included 3, 4, 9, 5, 10, and 4 complete trials, respectively, and a total of 16 participants (8 males and 8 females) contributed at least one complete trial (Table 1).

**Table 1:** Summary of participant demographics and experimental settings of dataset 1.

Attribute	Value
Number of participants	16 (8 males / 8 females)
Mean age (±SD)	25.1 ± 5.6 years
Mean body mass (±SD)	69.3 ± 10.8 kg
Mean height (±SD)	174.0 ± 7.3 cm
Mean body fat (±SD)	17.1 ± 6.1%
Clothing conditions	Permeable or impermeable coveralls
Environmental settings	**Setting 1**: 25 ${\;}^{\circ }$C, 50% RH, impermeable, no solar **Setting 2**: 40 ${\;}^{\circ }$C, 20% RH, permeable, no solar **Setting 3**: 40 ${\;}^{\circ }$C, 20% RH, impermeable, no solar **Setting 4**: 30 ${\;}^{\circ }$C, 35% RH, permeable, solar (530 W⋅m^−2^ on back) **Setting 5**: 40 ${\;}^{\circ }$C, 20% RH, permeable, solar (530 W⋅m^−2^ on back) **Setting 6**: 40 ${\;}^{\circ }$C, 35% RH, permeable, no solar
Activity protocol	**Setting 1**: 10 min rest → 40 min treadmill walking → rest (22 ${\;}^{\circ }$C, 50% RH) until rectal temperature drops by 0.4 ${\;}^{\circ }$C → 40 min treadmill walking. **Setting 6**: 10 min rest in climatic chamber → 60 min treadmill walking → rest (22 ${\;}^{\circ }$C, 50% RH) until 0.4 ${\;}^{\circ }$C drop in rectal temperature → 40 min treadmill walking. **Setting 2–5**: Same protocol as Setting 1.
Signals used for benchmark	Heart rate, rectal temperature, multi-site skin temperatures measured at the abdomen, calf, chest, foot, hand, head, lower arm, lower back, thigh, upper arm, and upper back.
Sampling interval	1 minute

Dataset 2 includes 13 male participants aged 18 to 45 years [60]. Each participant completed two heat-stress stages designed to impose different thermal load conditions. In each stage, participants first rested outdoors for 15 min, then indoors for another 15 min, followed by 20–60 min of indoor cycling performed under controlled heat stress. Exercise was terminated upon meeting one of the following criteria: a rectal temperature increase of at least 1.5${\;}^{\circ }$C above baseline, rectal temperature exceeding 38.5 ${\;}^{\circ }$C, voluntary exhaustion, or completion of 75 min of cycling. A final 30-minute outdoor rest period followed.

In Stage 1, participants wore light athletic clothing and cycled at 75% of maximal heart rate, whereas in Stage 2 they wore firefighter protective gear and cycled at 50% of ${\text{HR}}_{\max}$. A comprehensive set of physiological and environmental variables was collected throughout the protocol. These included heart rate, rectal temperature, and multi-site skin temperatures recorded at several anatomical locations. The original study additionally measured insulated skin temperatures and local skin heat-flux using specialized thermistors and heat-flux sensors; however, these experiment-specific measurements were excluded from the present benchmark analysis to maintain consistency with Dataset 1 and to ensure reliance on universally obtainable, non-insulated skin-temperature signals. Participant characteristics and environmental conditions are summarized in Table 2.

**Table 2:** Summary of participant demographics and experimental settings of dataset 2.

Attribute	Value
Number of participants	13 (13 males)
Mean age (±SD)	30.9 ± 5.4 years
Mean body mass (±SD)	77.5 ± 6.1 kg
Mean height (±SD)	179.2 ± 6.4 cm
Mean body fat (±SD)	13.1 ± 4.3%
Clothing conditions	T-shirts and shorts; firefighter protective gear
Environmental settings	35 ${\;}^{\circ }$C, 57% RH, no solar
Activity protocol	15 min rest (23 ${\;}^{\circ }$C, 25% RH) → 15 min rest (35 ${\;}^{\circ }$C, 57% RH) → 20–60 min cycling (35 ${\;}^{\circ }$C, 57% RH) →30 min rest (23 ${\;}^{\circ }$C, 25% RH)
Signals used for benchmark	Heart rate, rectal temperature, multi-site skin temperatures measured at the head, upper arm, forearm, hand, thigh, and calf.
Sampling interval	10 s

**Fig. 2:**
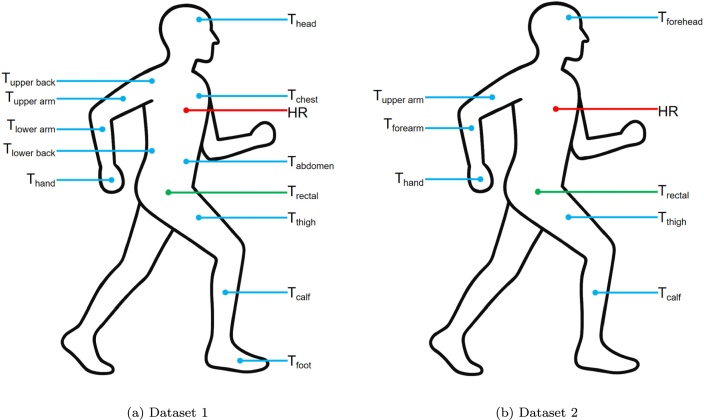
Schematic illustration of the anatomical measurement sites used for benchmark in Dataset 1 and Dataset 2. Only the signals included in the benchmark analysis are shown.

A schematic illustration of the measurement sites used for benchmark in Dataset 1 and Dataset 2 is provided in Fig. 2.

#### 2.2.2 Benchmark input signals

To ensure consistent and interpretable model comparison, all CBT-estimation algorithms were evaluated using a standardized set of input signals selected from each dataset. Only variables that were consistently available, non-invasive, and commonly obtainable in exercise or field monitoring contexts were included.

For Dataset 1, the benchmark inputs consisted of heart rate and multi-site skin temperatures recorded at eleven anatomical locations (abdomen, calf, chest, foot, hand, head, lower arm, lower back, thigh, upper arm, and upper back). For Dataset 2, the benchmark inputs included heart rate and multi-site skin temperatures measured at ten anatomical locations (head, upper arm, forearm, hand, thigh, calf, sternum, rib, scapula, and radial artery).

Rectal temperature served exclusively as the ground-truth reference in both datasets and was not used as a model input. Experiment-specific measurements—such as insulated skin temperatures and insulated climatic temperatures in Dataset 1, and heat-flux signals in Dataset 2—were excluded to maintain comparability across models and ensure practical relevance for real-world deployment.

Table 1, Table 2 list the exact benchmark signals used for each dataset.

#### 2.2.3 Evaluation framework

A standardized evaluation framework was implemented to ensure fair comparison across all CBT estimation models considered in the benchmark analysis. All signals used for the benchmark were used in their recorded form without additional smoothing, filtering, or interpolation, apart from the intrinsic synchronization performed during data acquisition. This ensured that no dataset-specific preprocessing biased the comparison between methods.

To provide a comprehensive assessment of model performance across representative heat-stress scenarios, all CBT-estimation methods were evaluated independently on Dataset 1 and Dataset 2. Each dataset constitutes a separate benchmark environment, and all comparisons were performed strictly within-dataset. This design yields two parallel evaluations—one for each dataset—ensuring that every model is assessed under identical conditions for a given dataset and allows consistent, head-to-head comparison across modeling approaches.

For all trainable models—including regression-based approaches, Kalman-filter variants, and machine-learning architectures—all parameters were re-estimated exclusively using the training folds of each dataset. Published coefficients from prior studies were not used, as those parameters were obtained under different populations, sensor configurations, and environmental conditions, and would therefore introduce dataset-specific bias and compromise the fairness of the benchmark.

A three-fold, trial-based cross-validation scheme was adopted. All complete trials from Dataset 1 and Dataset 2 were treated as independent units and partitioned into three mutually exclusive folds of approximately equal size. Trials were partitioned using a purely trial-based three-fold cross-validation scheme. In each cross-validation iteration, two folds were used for model training (and parameter estimation when required), while the remaining fold served as the held-out test set. This procedure was repeated three times such that each fold acted once as the test set.

For models that required a separate validation set (for example, to tune hyperparameters or implement early stopping), an internal validation subset was defined within the training data only. Specifically, a small proportion of the training trials (approximately 25%) was reserved as a validation set, while the remaining training trials were used for parameter fitting. The outer three-fold cross-validation structure and the test folds remained unchanged, ensuring that test data were never used for model selection or tuning.

All models were executed in a causal, forward-in-time manner to preserve real-time feasibility. Predictions were generated sequentially without access to future samples, and no retrospective smoothing or bidirectional filtering was applied. Because different models require different warm-up periods (e.g., minimum sequence length for recurrent architectures), performance metrics were computed only over the temporal intervals in which all models produced valid outputs. For each trial, the evaluation window was aligned by discarding the initial samples up to the maximum warm-up length across all models, ensuring that every model was evaluated over an identical, causally valid time segment. Ground-truth CBT and model-predicted CBT were then aligned sample-by-sample within this common evaluation window.

#### 2.2.4 Model inclusion criteria for benchmark

While the systematic review (Section 2.1) identified a wide range of core body temperature (CBT) estimation approaches, only a subset was eligible for inclusion in the standardized benchmark analysis. This distinction between reviewed models and benchmarked models was necessary to ensure methodological consistency, fair comparison, and practical relevance for real-world deployment.

Models were included in the benchmark comparison if they satisfied two feasibility criteria:

First, the model must rely exclusively on signals available in the standardized datasets used for benchmark, namely heart rate, non-invasive multi-site skin temperatures, and standard environmental parameters such as ambient temperature and relative humidity. Algorithms requiring experiment-specific or non–field-deployable measurements—for example heat flux, insulated skin temperature, or metabolic rate—were excluded to maintain a like-for-like comparison across methods and to reflect the constraints of wearable monitoring in operational settings.

Second, the original publication must provide a sufficiently detailed mathematical formulation or algorithmic description to permit re-implementation using the available inputs.

As a result, several studies identified in the systematic review—particularly those relying on heat flux or insulated skin temperature—were not included in the quantitative benchmark analysis due to input incompatibility. The benchmark comparison therefore focuses on algorithms that can be implemented using cardiovascular and thermometric signals commonly obtainable in exercise and heat-stress monitoring scenarios.

#### 2.2.5 Performance metrics

Model performance was evaluated using multiple complementary metrics that capture pointwise accuracy, agreement with the reference measurement, temporal tracking fidelity, and heat-strain detection capability. Pointwise accuracy was quantified using the root mean square error (RMSE) and mean absolute error (MAE). Agreement between predicted and reference CBT was assessed using Bland–Altman statistics, including the mean difference (MD, prediction-true) and standard deviation of differences (SD). The coefficient of determination ($R^{2}$) was used to quantify the proportion of variance explained by each model.

To assess the ability to identify physiologically meaningful elevations in CBT, a threshold of 38.0 ${\;}^{\circ }$C was applied in accordance with occupational heat-stress guidelines [1]. Binary classification performance was then assessed using the area under the ROC curve (AUC), accuracy, sensitivity, and specificity. All performance metrics were computed strictly within the aligned evaluation window described above.

## 3 Results

The database search identified a total of 12,535 records. After removal of duplicates, title and abstract screening, 183 full-text articles were assessed for eligibility. Of these, 38 studies met all inclusion criteria for the systematic review. The PRISMA flow diagram in Fig. 1 summarizes the identification, screening, exclusion, and final inclusion process.

Among the 38 included studies, 24 were excluded from the standardized benchmark analysis because their algorithms required incompatible inputs (e.g., heat flux, insulated skin temperature, metabolic rate, or detailed clothing insulation parameters). These excluded studies are listed in Table 3.

**Table 3:** Summary of studies included in the systematic review but excluded from the standardized benchmark analysis.

Study	Model type	Input variables	Reference CBT method	Reported metrics
Nazarian [53]	Kalman filter	Wrist air temperature; wrist skin temperature; humidity; heart rate	Gastrointestinal temperature	RMSE: 0.13 ${\;}^{\circ }$C; MAE: 0.10 ${\;}^{\circ }$C; MD: 0.008 ${\;}^{\circ }$C; SD: 0.27 ${\;}^{\circ }$C; $R^{2}$: 0.81
Moyen [62]	Hybrid model (machine-learning–based feature modeling + extended Kalman filter)	Heart rate; skin and ambient temperature(upper arm); skin and ambient humidity(upper arm); step rate; anthropometric data (age, height, body mass, sex)	Rectal temperature and gastrointestinal temperature	Rectal: RMSE: 0.30 ${\;}^{\circ }$C; MAE: 0.25 ${\;}^{\circ }$C; MD: 0.07 ${\;}^{\circ }$C; SD: 0.32 ${\;}^{\circ }$C.
				Gastrointestinal: RMSE: 0.29 ${\;}^{\circ }$C; MAE: 0.26 ${\;}^{\circ }$C; MD: 0.07 ${\;}^{\circ }$C; SD: 0.32 ${\;}^{\circ }$C.
Oleng’ [63]	Hybrid model (first-principles physiology model + autoregressive (AR) error-correction)	Core temperature history; metabolic rate; environmental conditions (air temperature, humidity, wind, radiant temperature); clothing insulation and thermal properties; body geometry and physical constants.	Rectal temperature	NR
Gribok [64]	Hybrid model (first-principles physiology model + autoregressive (AR) residual correction)	Core temperature history; metabolic rate; environmental conditions; clothing insulation parameters; physical constants	Rectal temperature (laboratory study); gastrointestinal temperature (field study)	20-min prediction RMSE (same-subject): $0.22\pm 0.04{\;}^{\circ }$C; (cross-subject): $0.23\pm 0.01{\;}^{\circ }$C
Hamatani [65]	Hybrid model (thermoregulation model (two-node) with parameter filtering)	Wrist skin temperature; heart rate; ambient temperature; humidity; solar radiation; clothing insulation; body anthropometrics	Rectal temperature	MD: −0.07 ${\;}^{\circ }$C
Laxminarayan [55]	Hybrid model (physics-based energy-balance + adaptive Kalman filter)	Heart rate; mean skin temperature; activity; ambient temperature; relative humidity	Rectal temperature	RMSE: 0.33 ${\;}^{\circ }$C (overall)
Tanaka [66]	Statistical model (linear regression)	Heart rate; exercise/recovery state	Rectal temperature	NR
Gibson [67]	Statistical model (linear regression with rate-of-change term)	Auditory canal temperature and its rate of change (dTc/dt)	Oesophageal temperature	RMSE: 0.11 ${\;}^{\circ }$C
Hansen [68]	Statistical model (multiple linear regression)	Tympanic temperature; aural canal temperature; cheek skin temperature	Rectal temperature	RMSE: 0.15 ${\;}^{\circ }$C (warm); 0.21 ${\;}^{\circ }$C (hot)
Gribok [69]	Statistical model (autoregressive with exogenous inputs)	Delayed core temperature; age; height; weight; body fat %; walking speed; mean radiant temperature; relative humidity	Rectal temperature	RMSE: ≈0.08 ${\;}^{\circ }$C (subject-specific); ≈0.19 ${\;}^{\circ }$C (cross-subject)
Gribok [70]	Statistical model (functional regression)	Heart rate; respiratory rate; skin temperature (not specified); subcutaneous glucose concentration	Gastrointestinal temperature	NR
Xu [41]	Statistical model (multiple linear regression)	Skin temperature and skin heat flux at six sites (forehead, sternum, pectoralis, rib, scapula, thigh)	Rectal temperature	$R^{2}$: 0.40 (forehead); 0.75 (sternum); 0.69 (scapula); 0.60 (pectoralis); 0.70 (rib); 0.59 (thigh); 0.79 (sternum + scapula + rib)
Richmond [42]	Statistical model (multiple linear regression)	Insulated neck skin temperature; microclimate temperature	Rectal temperature	RMSE: 0.20 ${\;}^{\circ }$C; $R^{2}$: 0.85
Niedermann [43]	Statistical model (multiple linear regression + PCA feature extraction)	Skin temperatures (upper arm, lower arm, thigh); skin heat flux (chest, back); heart rate	Gastrointestinal temperature	RMSE: 0.28 ${\;}^{\circ }$C (no radiation), $0.28\pm 0.03{\;}^{\circ }$C; $0.34{\;}^{\circ }$C (with radiation), $0.34\pm 0.07{\;}^{\circ }$C; MD: $0.04\pm 0.17{\;}^{\circ }$C (no radiation), $-0.17\pm 0.14{\;}^{\circ }$C (with radiation); SD: $0.17{\;}^{\circ }$C (no radiation), $0.14{\;}^{\circ }$C (with radiation); $R^{2}$: 0.72 (development), 0.70–0.73 (validation)
Richmond [58]	Statistical model (multiple linear regression)	Insulated neck skin temperature; microclimate temperature; heart rate (HR); work/rest indicator	Rectal temperature	RMSE: 0.24 ${\;}^{\circ }$C; $R^{2}$: 0.82
Seng [71]	Statistical model (nonlinear mixed-effects model)	Chest, upper arm, thigh, and calf skin temperature; load carried (kg); work–rest schedule; optional baseline core temperature data for updating	Gastrointestinal temperature	Population model: RMSE: 0.67 ${\;}^{\circ }$C; MD: −0.05 ${\;}^{\circ }$C; SD: 0.34 ${\;}^{\circ }$C.
				Mixed-effects Model 1 (Tc baseline only): RMSE: 0.57 ${\;}^{\circ }$C; MD: 0.07 ${\;}^{\circ }$C; SD: 0.29 ${\;}^{\circ }$C.
				Mixed-effects Model 2 (Tc baseline + Tc end-of-work): RMSE: 0.29 ${\;}^{\circ }$C; MD: 0.03 ${\;}^{\circ }$C; SD: 0.15 ${\;}^{\circ }$C
Gibson [72]	Statistical model (multiple linear regression)	Relative power; rating of perceived exertion; other exercise prescription variables	Rectal temperature	$R^{2}$: 0.625
Nakada [73]	Statistical model (multiple linear regression)	Three auditory canal temperatures	Oesophageal temperature	RMSE: 0.113 ${\;}^{\circ }$C; $R^{2}$: 0.904
Eggenberger [60]	Statistical model (PCA + multiple linear regression)	Heart rate; heat flux at sternum, scapula, and rib; insulated skin temperatures at sternum, scapula, rib, and radial artery; non-insulated skin temperatures at sternum, scapula, rib, radial artery, forehead, upper arm, forearm, hand, thigh, and calf	Rectal temperature	Max-input model (18 inputs): RMSE: 0.28 ${\;}^{\circ }$C; $R^{2}$: 0.703.
				Min-input model (2 inputs): RMSE: 0.29 ${\;}^{\circ }$C; $R^{2}$: 0.677
Tsadok [74]	Statistical model (curve fitting / regression models)	Core dual-sensor heat-flux temperatures (forehead and wrist)	Rectal temperature	RMSE: 0.29 ${\;}^{\circ }$C (forehead, overall), $0.23\pm 0.10{\;}^{\circ }$C (forehead, by subject); 0.40 ${\;}^{\circ }$C (wrist, overall), $0.31\pm 0.14{\;}^{\circ }$C (wrist, by subject); MAE: $0.20\pm 0.16{\;}^{\circ }$C (forehead), $0.27\pm 0.20{\;}^{\circ }$C (wrist); MD: $0.00\pm 0.25{\;}^{\circ }$C (forehead), $0.01\pm 0.32{\;}^{\circ }$C (wrist); SD: 0.25 ${\;}^{\circ }$C (forehead), 0.32 ${\;}^{\circ }$C (wrist)
Kurosaka [52]	Statistical model (multiple linear regression)	ECG-derived Poincaré plot features from current and previous time windows; previously estimated temperature change	Rectal temperature	RMSE: 0.023 ${\;}^{\circ }$C; SD: $\pm 0.13{\;}^{\circ }$C; $R^{2}$: 0.46
Li [75]	Statistical model (linear regression)	Skin temperature at multiple sites (chest, upper arm, thigh, calf); environmental parameters (air temperature, radiant temperature, humidity, air velocity)	Rectal temperature	No direct prediction accuracy metrics were reported (RMSE, MAE, MD, SD not provided).
Wang [76]	Machine learning model (random forest regression)	Sweat sodium concentration; sweat potassium concentration; heart rate; regional sweat rate	Gastrointestinal temperature	RMSE: 0.02 ${\;}^{\circ }$C
Tan [54]	Machine learning model (random forest regression)	Aural canal temperature; external auricle temperature; heart rate; trial phase information (baseline, exercise, recovery)	Gastrointestinal temperature	RMSE: 0.27 ${\;}^{\circ }$C; MAE: $0.20\pm 0.18{\;}^{\circ }$C; MD: $-0.02\pm 0.26{\;}^{\circ }$C; SD: 0.26 ${\;}^{\circ }$C

Note: NR: Not reported.

The remaining 14 studies satisfied all feasibility requirements for the benchmark. These models relied solely on signals available in Dataset 1 and Dataset 2 (i.e., heart rate, non-insulated multi-site skin temperatures, and standard environmental parameters) and provided sufficient methodological detail to allow re-implementation within the standardized evaluation framework. The benchmark-eligible models are summarized in Table 4.

**Table 4:** Summary of studies included in the systematic review and the standardized benchmark analysis.

Study	Model type	Model details	Input variables	Reference CBT method	Reported metrics	Continuous tracking metrics (Dataset 1)	Continuous tracking metrics (Dataset 2)	High-risk temperature detection (Dataset 1)	High-risk temperature detection (Dataset 2)
Buller [49]	Extended Kalman filter	Extended Kalman filter using heart rate as a nonlinear observation of core temperature. Time update assumes a linear autoregressive process model $CT_{t}=CT_{t-1}+f$, $f∼N(0,\gamma^{2})$. Heart rate is mapped to CT through a quadratic observation model $HR_{t}=b_{2}CT_{t}^{2}+b_{1}CT_{t}+b_{0}+g$, enabling EKF linearization and recursive minute-by-minute CT estimation.	Heart rate	Gastrointestinal temperature	RMSE: 0.30±0.13${\;}^{\circ }$C; MD: −0.03${\;}^{\circ }$C; SD: 0.32${\;}^{\circ }$C; $R^{2}$: 0.71	RMSE: $0.4108{\;}^{\circ }\text{C}$,MAE: $0.2943{\;}^{\circ }\text{C}$,MD: $-0.0520{\;}^{\circ }\text{C}$,SD: $0.4075{\;}^{\circ }\text{C}$,$R^{2}$: 0.4686.	RMSE: $0.6563{\;}^{\circ }\text{C}$,MAE: $0.4746{\;}^{\circ }\text{C}$,MD: $0.1388{\;}^{\circ }\text{C}$,SD: $0.6415{\;}^{\circ }\text{C}$,$R^{2}$: −0.4010.	Accuracy: 0.8186,Sensitivity: 0.7117,Specificity: 0.9171,AUC: 0.9004,RMSE: $0.5384{\;}^{\circ }\text{C}$.	Accuracy: 0.7728,Sensitivity: 0.3328,Specificity: 0.8549,AUC: 0.8202,RMSE: $0.4796{\;}^{\circ }\text{C}$.
Buller [77]	Extended Kalman filter	Same as Buller [49].	Heart rate	Gastrointestinal temperature	RMSE: 0.21±0.11${\;}^{\circ }$C; MD: 0.02${\;}^{\circ }$C; SD: 0.25${\;}^{\circ }$C	Same as [49]	Same as [49]	Same as [49]	Same as [49]
Hunt [78]	Extended Kalman filter	Same as Buller [49].	Heart rate	Gastrointestinal temperature	RMSE: 0.32${\;}^{\circ }$C; MAE: 0.25±0.20${\;}^{\circ }$C; MD: 0.01${\;}^{\circ }$C; SD: 0.33${\;}^{\circ }$C	Same as [49]	Same as [49]	Same as [49]	Same as [49]
Buller [79]	Extended Kalman filter	Same as Buller [49].	Heart rate	Gastrointestinal temperature	RMSE: 0.37±0.13${\;}^{\circ }$C (hot), 0.49±0.26${\;}^{\circ }$C (warm); MD: −0.10${\;}^{\circ }$C (hot), 0.34${\;}^{\circ }$C (warm); SD: 0.37${\;}^{\circ }$C (hot), 0.40${\;}^{\circ }$C (warm)	Same as [49]	Same as [49]	Same as [49]	Same as [49]
Egbert [50]	Extended Kalman filter	Same as Buller [49].	Heart rate	Gastrointestinal temperature	RMSE: 0.41${\;}^{\circ }$C; MAE: 0.32${\;}^{\circ }$C; MD: −0.14${\;}^{\circ }$C; SD: 0.39${\;}^{\circ }$C	Same as [49]	Same as [49]	Same as [49]	Same as [49]
Falcone [80]	Extended Kalman filter	Same as Buller [49].	Heart rate	Gastrointestinal temperature	Not reported in the paper.	Same as [49]	Same as [49]	Same as [49]	Same as [49]
Peggen [51]	Extended Kalman filter	Same as Buller [49].	Heart rate	Gastrointestinal temperature	RMSE: 0.37±0.15${\;}^{\circ }$C; MD: 0.09${\;}^{\circ }$C; SD: 0.22${\;}^{\circ }$C	Same as [49]	Same as [49]	Same as [49]	Same as [49]
Seng [45]	Extended Kalman filter	Nonlinear state-space EKF with heart-rate–driven dynamics and cubic skin-temperature observation.The state transition follows a nonlinear autoregressive model,$T_{c,t}=\alpha_{0}+\alpha_{1}T_{c,t-1}+\alpha_{2}T_{c,t-1}^{2}+\alpha_{3}HR_{t-1}+\omega_{t}$,and the observation model maps chest skin temperature to core temperature via a cubic function,$T_{sk,t}=\beta_{0}+\beta_{1}T_{c,t}+\beta_{2}T_{c,t}^{2}+\beta_{3}T_{c,t}^{3}+\eta_{t}$.	Surface chest skin temperature; heart rate	Gastrointestinal temperature	RMSE: 0.29${\;}^{\circ }$C; MD: 0.11${\;}^{\circ }$C; SD: 0.27${\;}^{\circ }$C	RMSE: $0.3269{\;}^{\circ }\text{C}$; MAE: $0.2530{\;}^{\circ }\text{C}$; MD: $-0.0214{\;}^{\circ }\text{C}$; SD: $0.3262{\;}^{\circ }\text{C}$; $R^{2}$: 0.6634	RMSE: $0.5220{\;}^{\circ }\text{C}$; MAE: $0.4122{\;}^{\circ }\text{C}$; MD: $0.1466{\;}^{\circ }\text{C}$; SD: $0.5009{\;}^{\circ }\text{C}$; $R^{2}$: 0.1139	Accuracy: 0.8237; Sensitivity: 0.7742; Specificity: 0.8693; AUC: 0.9201; RMSE: $0.3981{\;}^{\circ }\text{C}$	Accuracy: 0.7904; Sensitivity: 0.8127; Specificity: 0.7862; AUC: 0.8740; RMSE: $0.3903{\;}^{\circ }\text{C}$
Rizvi [46]	Extended Kalman filter	Modified EKF with phase-dependent measurement models. Time update: $CT_{t}=a1*CT_{t-1}+a0+\varepsilon$. HR-based observation: $HR_{t}=b_{1}CT_{t}^{2}+b_{2}CT_{t}+b_{3}+\eta$, with separate $\left(b_{1},b_{2},b_{3}\right)$ for exercise and recovery, selected via a 5-min moving-average HR rule.	Heart rate	Gastrointestinal temperature	RMSE: 0.16${\;}^{\circ }$C; MD: −0.03${\;}^{\circ }$C; SD: 0.16${\;}^{\circ }$C	RMSE: $0.3952{\;}^{\circ }\text{C}$; MAE: $0.3079{\;}^{\circ }\text{C}$; MD: $-0.0172{\;}^{\circ }\text{C}$; SD: $0.3948{\;}^{\circ }\text{C}$; $R^{2}$: 0.5082	RMSE: $0.5400{\;}^{\circ }\text{C}$; MAE: $0.4279{\;}^{\circ }\text{C}$; MD: $0.0472{\;}^{\circ }\text{C}$; SD: $0.5379{\;}^{\circ }\text{C}$; $R^{2}$: 0.0517	Accuracy: 0.8186; Sensitivity: 0.7523; Specificity: 0.8797; AUC: 0.9028; RMSE: $0.4932{\;}^{\circ }\text{C}$	Accuracy: 0.8144; Sensitivity: 0.7180; Specificity: 0.8324; AUC: 0.8778; RMSE: $0.5062{\;}^{\circ }\text{C}$
Falcone [81]	Extended Kalman filter	A biphasic Kalman filter (BKFB) model with separate observation functions for core-temperature increase and decrease phases. The time-update assumes $CT_{t}=a1*CT_{t-1}+a0+\omega_{t}$, while the measurement model maps heart rate (HR) to core temperature through phase-specific quadratic functions, ${\text{HR}}_{t}=f_{↑}(CT_{t})+\eta_{t}$ during activity and ${\text{HR}}_{t}=f_{↓}(CT_{t})+\eta_{t}$ during recovery, where $f_{↑}$ and $f_{↓}$ are distinct second-order polynomials. Phase switching is governed by a heart-rate threshold rule.	Heart rate	Gastrointestinal temperature	RMSE: 0.28±0.12${\;}^{\circ }$C	RMSE: $0.3662{\;}^{\circ }\text{C}$; MAE: $0.2700{\;}^{\circ }\text{C}$; MD: $-0.0170{\;}^{\circ }\text{C}$; SD: $0.3658{\;}^{\circ }\text{C}$; $R^{2}$: 0.5777	RMSE: $0.4921{\;}^{\circ }\text{C}$; MAE: $0.3742{\;}^{\circ }\text{C}$; MD: $-0.0309{\;}^{\circ }\text{C}$; SD: $0.4911{\;}^{\circ }\text{C}$; $R^{2}$: 0.2124	Accuracy: 0.8270; Sensitivity: 0.7373; Specificity: 0.9096; AUC: 0.9172; RMSE: $0.4808{\;}^{\circ }\text{C}$	Accuracy: 0.8478; Sensitivity: 0.4567; Specificity: 0.9208; AUC: 0.8196; RMSE: $0.7072{\;}^{\circ }\text{C}$
Buller [82]	Kalman filter	A linear Kalman filter was implemented with core temperature (CT) as the latent state. CT dynamics were modeled using a first-order autoregressive process:$CT_{t}=a_{1}CT_{t-1}+a_{0}+\omega_{t},$Heart rate (HR) was incorporated through a linear observation model derived from two anchor points in the HR–CT plane:$HR_{t}=a\;CT_{t}+b+\nu_{t},$	Heart rate	Gastrointestinal temperature	RMSE: 0.30${\;}^{\circ }$C; MD: 0.02${\;}^{\circ }$C; SD: 0.18${\;}^{\circ }$C	RMSE: $0.4053{\;}^{\circ }\text{C}$; MAE: $0.3530{\;}^{\circ }\text{C}$; MD: $0.2693{\;}^{\circ }\text{C}$; SD: $0.3029{\;}^{\circ }\text{C}$; $R^{2}$: 0.4827	RMSE: $0.4712{\;}^{\circ }\text{C}$; MAE: $0.3914{\;}^{\circ }\text{C}$; MD: $0.0039{\;}^{\circ }\text{C}$; SD: $0.4712{\;}^{\circ }\text{C}$; $R^{2}$: 0.2778	Accuracy: 0.7065; Sensitivity: 0.9744; Specificity: 0.4600; AUC: 0.9215; RMSE: $0.3146{\;}^{\circ }\text{C}$	Accuracy: 0.6690; Sensitivity: 0.8411; Specificity: 0.9772; AUC: 0.7486; RMSE: $0.6690{\;}^{\circ }\text{C}$
Welles [83]	Kalman filter	A linear Kalman filter was used to estimate core temperature (CT) from head skin temperature ($T_{\text{sk}}$) and heart rate (HR). The state-transition model followed a first-order autoregressive form:$CT_{t}=a\;CT_{t-1}+a_{0}+w_{t},$The observation model linearly mapped CT to the measured signals:$z_{t}=\left[\begin{array}{c} T_{\text{sk}}(t)\\ HR(t) \end{array}\right]=C\;CT_{t}+b+v_{t},$	Skin temperature (forehead, pectoralis, scapula, lower back, vastus medialis, gastrocnemius); heart rate	Gastrointestinal temperature	Thigh: RMSE(overall)=0.30 ${\;}^{\circ }$C; RMSE(by subject)=0.28±0.10 ${\;}^{\circ }$C; MD=0.04 ${\;}^{\circ }$C, SD=0.19 ${\;}^{\circ }$C.Scapula: RMSE(overall)=0.28 ${\;}^{\circ }$C; RMSE(by subject)=0.25±0.12 ${\;}^{\circ }$C; MD=−0.03 ${\;}^{\circ }$C, SD=0.15 ${\;}^{\circ }$C.Forehead: RMSE(overall)=0.30 ${\;}^{\circ }$C; RMSE(by subject)=0.28±0.12 ${\;}^{\circ }$C; MD=0.04 ${\;}^{\circ }$C, SD=0.17 ${\;}^{\circ }$C.	Thigh: RMSE: $0.3618{\;}^{\circ }\text{C}$; MAE: $0.2849{\;}^{\circ }\text{C}$; MD: $-0.0203{\;}^{\circ }\text{C}$; SD: $0.3612{\;}^{\circ }\text{C}$; $R^{2}$: 0.5877; Forehead: RMSE: $0.4489{\;}^{\circ }\text{C}$; MAE: $0.3284{\;}^{\circ }\text{C}$; MD: $-0.0368{\;}^{\circ }\text{C}$; SD: $0.4474{\;}^{\circ }\text{C}$; $R^{2}$: 0.3654	Thigh: RMSE: $0.6799{\;}^{\circ }\text{C}$; MAE: $0.5440{\;}^{\circ }\text{C}$; MD: $0.1557{\;}^{\circ }\text{C}$; SD: $0.6618{\;}^{\circ }\text{C}$; $R^{2}$: −0.5035; Forehead: RMSE: $0.5348{\;}^{\circ }\text{C}$; MAE: $0.4156{\;}^{\circ }\text{C}$; MD: $-0.0222{\;}^{\circ }\text{C}$; SD: $0.5343{\;}^{\circ }\text{C}$; $R^{2}$: 0.0698	Thigh: Accuracy: 0.8273; Sensitivity: 0.7986; Specificity: 0.8538; AUC: 0.9023; RMSE: $0.3858{\;}^{\circ }\text{C}$; Forehead: Accuracy: 0.7911; Sensitivity: 0.6792; Specificity: 0.8941; AUC: 0.8749; RMSE: $0.5864{\;}^{\circ }\text{C}$	Thigh: Accuracy: 0.7506; Sensitivity: 0.7744; Specificity: 0.7461; AUC: 0.8233; RMSE: $0.5981{\;}^{\circ }\text{C}$; Forehead: Accuracy: 0.7855; Sensitivity: 0.4421; Specificity: 0.8496; AUC: 0.8107; RMSE: $0.5318{\;}^{\circ }\text{C}$
Kim [84]	Statistical model (linear regression)	Rectal temperature predicted from single-site skin temperature using site-specific linear regression, $CT=a\;T_{\text{skin}}+b$.	Twelve skin temperatures at forehead, chest, abdomen, upper back, upper arm, forearm, hand, thigh, calf, dorsal foot, lateral foot, and toe	Rectal temperature	$R^{2}$: 0.826 (forehead); 0.824 (chest); 0.473 (abdomen); 0.494 (back); 0.779 (upper arm); 0.753 (forearm); 0.382 (hand); 0.688 (thigh); 0.619 (calf); 0.684 (foot); 0.606 (lateral foot); 0.571 (toe)	Forehead: RMSE: $0.5701{\;}^{\circ }\text{C}$; Chest: RMSE: $0.5462{\;}^{\circ }\text{C}$; Abdomen: RMSE: $0.5450{\;}^{\circ }\text{C}$; Upper back: RMSE: $0.5708{\;}^{\circ }\text{C}$; Lower back: RMSE: $0.5638{\;}^{\circ }\text{C}$; Upper arm: RMSE: $0.5068{\;}^{\circ }\text{C}$; Lower arm: RMSE: $0.5264{\;}^{\circ }\text{C}$; Hand: RMSE: $0.5425{\;}^{\circ }\text{C}$; Thigh: RMSE: $0.4995{\;}^{\circ }\text{C}$; Calf: RMSE: $0.5093{\;}^{\circ }\text{C}$; Foot: RMSE: $0.4216{\;}^{\circ }\text{C}$	Forehead: RMSE: $0.5107{\;}^{\circ }\text{C}$; Upper arm: RMSE: $0.5444{\;}^{\circ }\text{C}$; Lower arm: RMSE: $0.5521{\;}^{\circ }\text{C}$; Hand: RMSE: $0.5473{\;}^{\circ }\text{C}$; Thigh: RMSE: $0.5259{\;}^{\circ }\text{C}$; Calf: RMSE: $0.5304{\;}^{\circ }\text{C}$	Forehead: Sensitivity: 0.2989; Specificity: 0.7827; Chest: Sensitivity: 0.4953; Specificity: 0.6789; Abdomen: Sensitivity: 0.5447; Specificity: 0.6834; Upper back: Sensitivity: 0.2226; Specificity: 0.7765; Lower back: Sensitivity: 0.3271; Specificity: 0.7720; Upper arm: Sensitivity: 0.6417; Specificity: 0.6580; Lower arm: Sensitivity: 0.5610; Specificity: 0.6400; Hand: Sensitivity: 0.5622; Specificity: 0.6247; Thigh: Sensitivity: 0.6467; Specificity: 0.6620; Calf: Sensitivity: 0.6467; Specificity: 0.6710; Foot: Sensitivity: 0.8236; Specificity: 0.6394	Forehead: Sensitivity: 0.0000; Specificity: 0.9971; Upper arm: Sensitivity: 0.0000; Specificity: 1.0000; Lower arm: Sensitivity: 0.0000; Specificity: 1.0000; Hand: Sensitivity: 0.0000; Specificity: 1.0000; Thigh: Sensitivity: 0.0000; Specificity: 1.0000; Calf: Sensitivity: 0.0000; Specificity: 1.0000
Han [85]	Machine learning model (Kalman filter + deep learning, LTSF-Informer)	Hybrid framework combining an extended Kalman filter with a long-term sequence forecasting model (Informer). The EKF estimates CT from HR using $CT_{t}=a_{1}CT_{t-1}+a_{0}+f_{t}$ and $HR_{t}=b_{2}CT_{t}^{2}+b_{1}CT_{t}+b_{0}+g_{t}$, providing a denoised HR-derived CT feature. The Informer then predicts future CT using ProbSparse attention and encoder distillation; the combined model outputs next-step CT forecasts.	Heart rate; 7-point skin temperature (forehead, chest, forearm, hand, thigh, calf, foot); mean skin temperature	Gastrointestinal temperature	HR only: MAE: 0.17${\;}^{\circ }$C; RMSE: 0.28${\;}^{\circ }$C; $R^{2}$: 0.70.				
					HR + 3-point T_sk_: MAE: 0.13${\;}^{\circ }$C; RMSE: 0.24${\;}^{\circ }$C; $R^{2}$: 0.72.				
					HR + 4-point T_sk_: MAE: 0.11${\;}^{\circ }$C; RMSE: 0.18${\;}^{\circ }$C; $R^{2}$: 0.83.				
					HR + 7-point T_sk_: MAE: 0.09${\;}^{\circ }$C; RMSE: 0.12${\;}^{\circ }$C; $R^{2}$: 0.92	HR only: RMSE: $0.4107{\;}^{\circ }\text{C}$; MAE: $0.3181{\;}^{\circ }\text{C}$; MD: $-0.0737{\;}^{\circ }\text{C}$; SD: $0.4040{\;}^{\circ }\text{C}$; $R^{2}$: 0.1991; HR + 3-point Tsk: RMSE: $0.4128{\;}^{\circ }\text{C}$; MAE: $0.3187{\;}^{\circ }\text{C}$; MD: $-0.0985{\;}^{\circ }\text{C}$; SD: $0.4008{\;}^{\circ }\text{C}$; $R^{2}$: 0.1911; HR + 4-point Tsk: RMSE: $0.4110{\;}^{\circ }\text{C}$; MAE: $0.3166{\;}^{\circ }\text{C}$; MD: $-0.0990{\;}^{\circ }\text{C}$; SD: $0.3989{\;}^{\circ }\text{C}$; $R^{2}$: 0.1982; HR + 7-point Tsk: RMSE: $0.4152{\;}^{\circ }\text{C}$; MAE: $0.3211{\;}^{\circ }\text{C}$; MD: $-0.0993{\;}^{\circ }\text{C}$; SD: $0.4031{\;}^{\circ }\text{C}$; $R^{2}$: 0.1816	HR only: RMSE: $0.3354{\;}^{\circ }\text{C}$; MAE: $0.2708{\;}^{\circ }\text{C}$; MD: $0.0289{\;}^{\circ }\text{C}$; SD: $0.3341{\;}^{\circ }\text{C}$; $R^{2}$: 0.5949	HR only: Accuracy: 0.7301; Sensitivity: 0.8080; Specificity: 0.6138; AUC: 0.7987; RMSE: $0.4483{\;}^{\circ }\text{C}$; HR + 3-point Tsk: Accuracy: 0.7316; Sensitivity: 0.8856; Specificity: 0.5019; AUC: 0.7968; RMSE: $0.4652{\;}^{\circ }\text{C}$; HR + 4-point Tsk: Accuracy: 0.7372; Sensitivity: 0.8881; Specificity: 0.5121; AUC: 0.8040; RMSE: $0.4627{\;}^{\circ }\text{C}$; HR + 7-point Tsk: Accuracy: 0.7271; Sensitivity: 0.8856; Specificity: 0.4907; AUC: 0.7915; RMSE: $0.4672{\;}^{\circ }\text{C}$	HR only: Accuracy: 0.8533; Sensitivity: 0.0000; Specificity: 1.0000; AUC: 0.8343; RMSE: $0.4898{\;}^{\circ }\text{C}$

Note: Due to space constraints, for Kim [84] only RMSE, sensitivity and specificity are reported in the table. NR: Not reported; $CT_{t}$: core temperature at time t; $HR_{t}$: heart rate at time t; $T_{\text{sk},t}$: skin temperature at time t; y(t): core-temperature time series used in autoregressive modeling; ${\hat{CT}}_{t+1}$: one-step-ahead predicted core temperature; ${\tilde{CT}}_{t}$: filtered or smoothed core-temperature signal; $z_{t}$: observation vector at time t; f, $f_{t}$, g, $g_{t}$: random noise terms in the process or observation models; $a_{i}$, $b_{i}$, $c_{i}$: model or regression coefficients; $w_{t}$, $v_{t}$, $\nu_{t}$, $\varepsilon_{t}$, $\eta_{t}$, $\omega_{t}$: process or measurement noise terms.

Complete study characteristic summaries, including the detailed quality-related descriptive information (e.g., participant profiles, experimental settings, sensor configurations), are provided in the supplementary materials.

### 3.1 Model categories and inputs

**Fig. 3:**
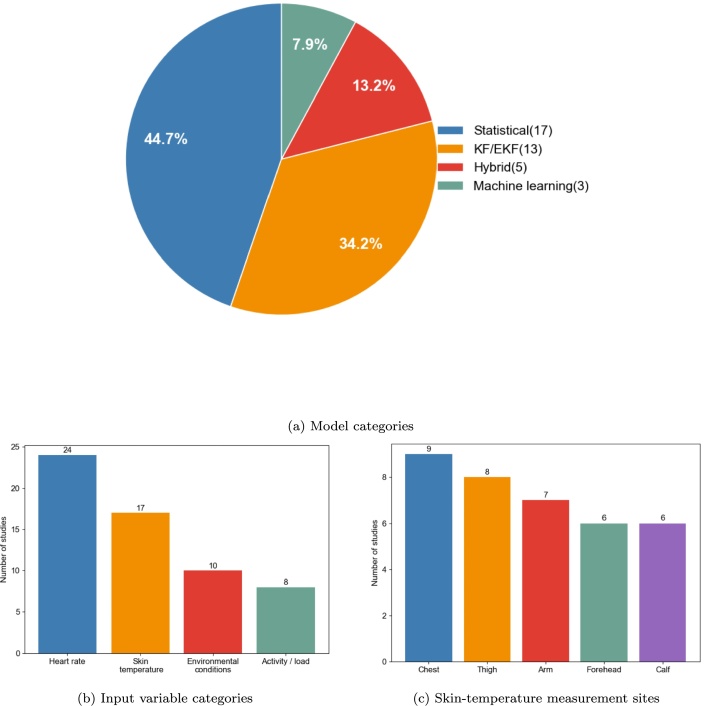
Overview of modeling approaches and input characteristics across the 38 studies included in the systematic review. (a) Distribution of the four modeling categories: EKF/KF models, statistical models, pure ML models, and hybrid physiological–data-driven models. (b) Five most frequently used input variable categories. (c) Five most frequently reported anatomical skin-temperature sites.

Across the 38 studies included in the systematic review, four modeling categories were identified: (1) Kalman filter or extended Kalman filter (EKF/KF) models, (2) statistical models, (3) pure machine-learning (ML) models, and (4) hybrid models integrating physiological or biophysical formulations with data-driven components. Statistical models were the most common (17 studies), followed by EKF/KF approaches (13 studies). Hybrid models were reported in 5 studies, and 3 studies used pure ML methods. The distribution of these four method categories is shown in Fig. 3 (a).

Most EKF/KF-based approaches implemented the canonical heart-rate–driven extended Kalman filter in which core temperature is treated as a latent state with an autoregressive time update and heart rate is linked to core temperature through the observation function. Variants of this framework included cubic state space EKF formulations using chest skin temperature as the primary observation, phase-dependent EKFs with separate exercise and recovery measurement models, and biphasic Kalman filters with distinct observation functions for heating and cooling phases. In addition to these extended Kalman filters, several studies used linear Kalman filters with either heart rate and skin temperature or core temperature history as the primary inputs.

Statistical models spanned a broad spectrum of regression and time-series formulations. Frequently used approaches included multiple linear regression linking core temperature to one or more skin temperatures or tympanic/aural measurements, and regression frameworks augmented with principal component analysis. Other statistical models comprised nonlinear mixed-effects models incorporating individual baseline or end-of-work core temperature, functional regression using multiple physiological inputs, and curve-fitting models applied to dual-sensor heat flux measurements.

Pure ML models were less frequently reported and mainly consisted of ensemble and deep learning methods. Two studies employed random forest regression using combinations of cardiovascular, thermometric, and sweat composition variables, whereas one study used a long-term sequence forecasting architecture (Informer) that consumed heart rate and multi-site skin temperatures, either directly or in combination with features derived from a preceding Kalman filter. Hybrid models, in contrast, explicitly combined physics-based or thermophysiological formulations with data-driven components—for example, first-principles thermoregulation or energy balance models with autoregressive error-correction, or two-node thermoregulation models with parameter filtering and adaptive Kalman filtering.

The frequency of input variables varied across studies. The five most commonly used input categories were heart rate (24 studies), skin temperature (17 studies), environmental conditions (10 studies), and load- or activity-related variables (8 studies). These counts are summarized in Fig. 3(b).

Among studies using skin temperature, the five most frequently reported anatomical measurement sites were the chest (9 studies), thigh (8 studies), arm (7 studies), forehead (6 studies), and calf (6 studies). These distributions are illustrated in Fig. 3(c).

Across the included studies, validated internal core-temperature measurements were used as the reference standard. Gastrointestinal telemetry pills were the most frequently employed reference (19 studies), followed by rectal thermometry (15 studies). Two studies used oesophageal temperature, and two studies reported both rectal and gastrointestinal measurements.

### 3.2 Experiment settings

**Fig. 4:**
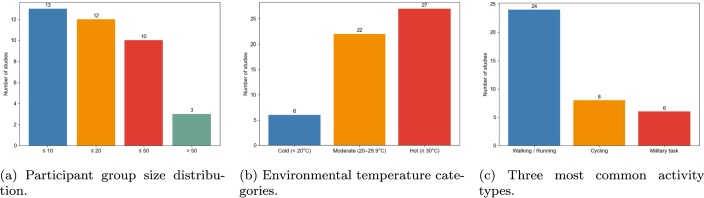
Overview of study characteristics across the included studies. (a) Participant group size distribution (all 38 studies). (b) Environmental exposure categories (Cold <20 ${\;}^{\circ }$C, Moderate 20–29.9 ${\;}^{\circ }$C, hot ≥30 ${\;}^{\circ }$C). One study did not report ambient temperature and is therefore not represented in panel (b). (c) Distribution of the three most frequently reported activity types (walking/running, military tasks, and cycling). Categories in (b) and (c) are not mutually exclusive, so a single study may contribute to multiple categories.

Across the 38 included studies, substantial heterogeneity was observed in participant sample sizes, environmental exposures, and activity protocols (Fig. 4).

Participant group size varied widely (Fig. 4 (a)). A total of 13 studies enrolled ≤ 10 participants, 12 studies included 11–20 participants, 10 studies included 21–50 participants, and only 3 studies enrolled more than 50 participants.

Environmental conditions also showed wide variability (Fig. 4 (b)). Using three temperature bands: Cold (<20 ${\;}^{\circ }$C), Moderate (20–29.9 ${\;}^{\circ }$C), and Hot (≥30 ${\;}^{\circ }$C), we found that 6 studies included at least one cold-temperature protocol, 22 studies included moderate-temperature conditions, and 27 studies involved hot environments. A single study did not report ambient temperature and was excluded from this figure.

A similarly diverse range of activity protocols was observed (Fig. 4 (c)). Walking or running was the most frequently employed modality (24 studies), followed by military or load-carriage tasks (9 studies) and cycling protocols (8 studies). Because these categories are not mutually exclusive, individual studies could contribute to multiple activity types or temperature bands.

### 3.3 Reported performance across studies

Across the 38 included studies, the performance metrics reported in the original publications showed substantial heterogeneity. For the purpose of this systematic review, only five commonly reported accuracy measures were extracted: root mean square error (RMSE), mean absolute error (MAE), mean difference (MD), standard deviation of error (SD), and the coefficient of determination ($R^{2}$). These were the only metrics that appeared with sufficient frequency across studies to permit consistent extraction.

However, the level of detail varied considerably. Several studies reported full sets of overall error metrics (e.g., RMSE, MAE, MD, and SD), whereas others provided only a subset (e.g., reporting RMSE alone or $R^{2}$ only). A number of studies also reported site-specific or condition-specific values apart from the overall metrics, and some studies did not provide any numerical performance values suitable for extraction.

Among the models that reported RMSE, values ranged from as low as approximately 0.02 ${\;}^{\circ }$C (e.g., sweat–electrolyte–based random forest models) to over 0.60 ${\;}^{\circ }$C in population-level mixed-effects models evaluated under field or occupational conditions.

Kalman filter and extended Kalman filter (EKF) studies generally reported RMSE values in the range of 0.13–0.41 ${\;}^{\circ }$C, depending on the environmental setting, activity protocol, and whether phase-specific models (e.g., activity vs. recovery) or additional inputs such as skin temperature.

Statistical models exhibited similarly wide variability. Multiple regression approaches, which typically incorporated combinations of skin temperatures, heat-flux measurements, or environmental parameters, demonstrated substantial performance differences across studies. Depending on the specific anatomical sites included, the model formulation (e.g., PCA-based feature reduction or nonlinear mixed-effects structures), the presence or absence of heat-flux inputs, and additional specialized variables (e.g., insulated skin temperatures or ECG-derived Poincaré features), reported RMSE values ranged from 0.02 ${\;}^{\circ }$C to 0.67 ${\;}^{\circ }$C. This broad spread reflects heterogeneity in measurement site selection, the number and type of included physiological signals, and the diversity of environmental conditions and activity protocols.

Machine-learning approaches were less widely represented but exhibited performance ranges comparable to those of the statistical models. Two studies employing random forest regression reported markedly different performance depending on the input composition. When sweat-based biomarkers were included together with heart rate, RMSE values as low as 0.02 ${\;}^{\circ }$C were observed, whereas models relying on aural or auricle temperature together with heart rate reported RMSE values around 0.27 ${\;}^{\circ }$C. The only deep-learning-based formulation—a long-term sequence forecasting model (Informer) combined with or without EKF-derived features—showed a strong dependence on the number of skin-temperature sites used as input. Reported RMSE values ranged from approximately 0.28 ${\;}^{\circ }$C for heart-rate-only models to 0.12 ${\;}^{\circ }$C when seven-site skin-temperature arrays were included. These findings indicate that ML model performance is highly sensitive to input dimensionality and physiological signal diversity.

Hybrid models, which combine physics-based thermophysiological formulations with data-driven components, demonstrate similarly broad performance variability. Models incorporating first-principles thermoregulation or energy-balance equations together with autoregressive residual correction achieve RMSE values of approximately 0.22–0.30 ${\;}^{\circ }$C in laboratory and field evaluations. Energy-balance formulations combined with adaptive Kalman filtering show comparable performance, with reported RMSE values around 0.33 ${\;}^{\circ }$C. Others that integrate two-node thermoregulation models with parameter filtering report small mean differences (e.g., –0.07 ${\;}^{\circ }$C) but do not provide full accuracy metrics.

### 3.4 Benchmark performance across the eligible studies

**Fig. 5:**
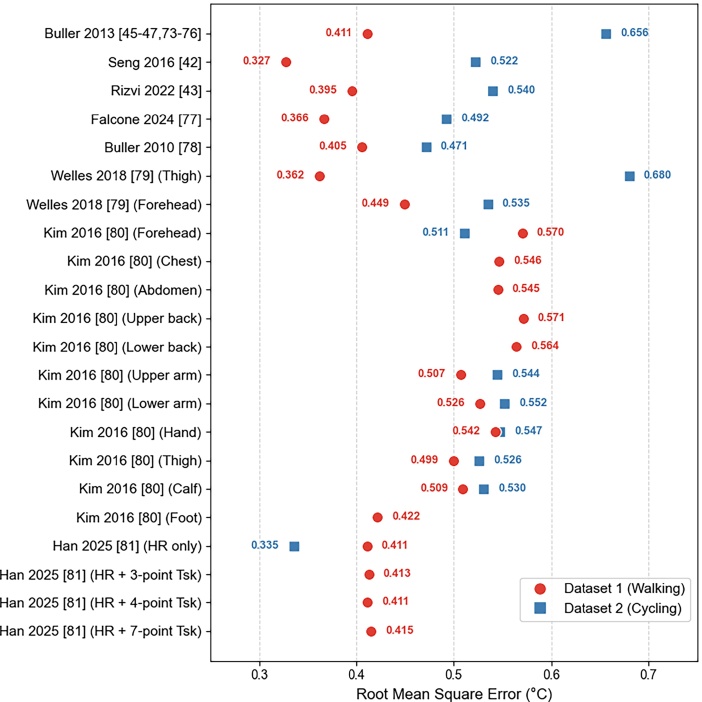
Benchmark comparison of continuous core body temperature tracking accuracy in selected studies, quantified by RMSE.

Fig. 5 summarizes the benchmarked continuous CBT tracking accuracy of all 14 selected studies on Dataset 1 and Dataset 2, quantified by RMSE. For each model, detailed performance is provided in Table 4.

Across studies adopting the HR–only extended Kalman filter (EKF) originally proposed by Buller et al. [49], multiple publications reported RMSE values under different experimental conditions. Buller et al. [49] reported an RMSE of $0.30\pm 0.13{\;}^{\circ }\text{C}$, while subsequent evaluations reported an RMSE of $0.21\pm 0.11{\;}^{\circ }\text{C}$ [77], $0.32{\;}^{\circ }\text{C}$ [78], $0.37\pm 0.13{\;}^{\circ }\text{C}$ in hot conditions and $0.49\pm 0.26{\;}^{\circ }\text{C}$ in warm conditions [79], $0.41{\;}^{\circ }\text{C}$ [50], and $0.37\pm 0.15{\;}^{\circ }\text{C}$ [51]. Under the standardized benchmark framework, the same HR-only EKF formulation yielded an RMSE of $0.4108{\;}^{\circ }\text{C}$ on Dataset 1 and $0.6563{\;}^{\circ }\text{C}$ on Dataset 2.

For EKF models incorporating skin temperature observations, the nonlinear EKF proposed by Seng et al. [45], which uses chest skin temperature and HR as inputs, achieved a benchmark RMSE of $0.3269{\;}^{\circ }\text{C}$ on Dataset 1 and $0.5220{\;}^{\circ }\text{C}$ on Dataset 2. In the original study, Seng et al. [45] reported an RMSE of $0.29{\;}^{\circ }\text{C}$.

Phase-dependent EKF variants showed slightly improved benchmark RMSE values compared with HR–only EKF approaches. The modified EKF reported by Rizvi [46] achieved an RMSE of $0.3952{\;}^{\circ }\text{C}$ on Dataset 1 and $0.5400{\;}^{\circ }\text{C}$ on Dataset 2, compared with a reported RMSE of $0.16{\;}^{\circ }\text{C}$. The biphasic Kalman filter model proposed by Falcone et al. [81] yielded a benchmark RMSE of $0.3662{\;}^{\circ }\text{C}$ on Dataset 1 and $0.4921{\;}^{\circ }\text{C}$ on Dataset 2, whereas the original publication reported an RMSE of $0.28\pm 0.12{\;}^{\circ }\text{C}$.

Linear Kalman filter models combining heart rate with skin temperature inputs demonstrated site-dependent benchmark performance. The linear KF reported by Buller et al. [82] achieved an RMSE of $0.4053{\;}^{\circ }\text{C}$ on Dataset 1 and $0.4712{\;}^{\circ }\text{C}$ on Dataset 2, compared with a reported RMSE of $0.30{\;}^{\circ }\text{C}$. For the linear KF proposed by Welles et al. [83], benchmark RMSE on Dataset 1 ranged from $0.3618{\;}^{\circ }\text{C}$ for the thigh-based configuration to $0.4489{\;}^{\circ }\text{C}$ for the forehead-based configuration, while on Dataset 2 the corresponding RMSE values were $0.6799{\;}^{\circ }\text{C}$ and $0.5348{\;}^{\circ }\text{C}$. In the original study, reported overall RMSE values ranged from 0.28 to $0.30{\;}^{\circ }\text{C}$ depending on the anatomical site.

Single-site statistical regression models based on skin temperature showed higher benchmark RMSE values. The linear regression models reported by Kim [84] yielded Dataset 1 RMSE values ranging from $0.4216{\;}^{\circ }\text{C}$ to $0.5708{\;}^{\circ }\text{C}$ across anatomical locations, with Dataset 2 RMSE values ranging from $0.5107{\;}^{\circ }\text{C}$ to $0.5521{\;}^{\circ }\text{C}$. The original study reported site-specific $R^{2}$ values but did not report RMSE or MAE.

For the hybrid deep-learning EKF–Informer model proposed by Han et al. [85], the benchmarked heart-rate–only variant achieved an RMSE of $0.4107{\;}^{\circ }\text{C}$ on Dataset 1 and $0.3354{\;}^{\circ }\text{C}$ on Dataset 2. In the original study, the reported RMSE for the heart-rate–only variant was $0.28{\;}^{\circ }\text{C}$, with lower reported RMSE values observed when additional multi-site skin temperature inputs were included.

**Fig. 6:**
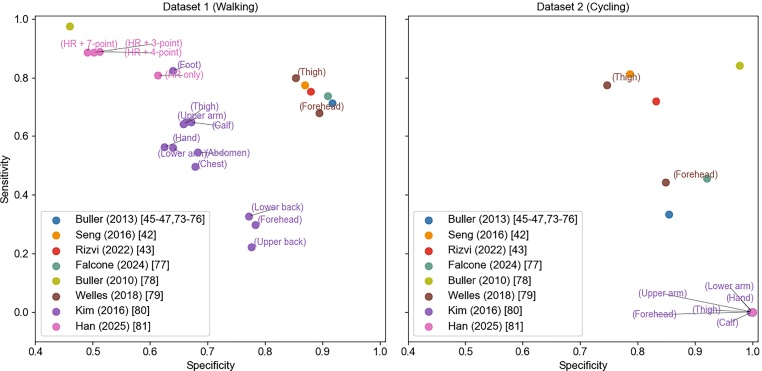
Sensitivity and specificity for high-risk core temperature detection (≥38.0 ${\;}^{\circ }$C) across selected studies.

Fig. 6 summarizes sensitivity and specificity for detecting elevated CBT using a threshold of $38.0{\;}^{\circ }\text{C}$ in Dataset 1 and Dataset 2.

On Dataset 1, heart-rate–only EKF implementations yielded sensitivity values ranging from 0.7117 to 0.7523, with corresponding specificity values between 0.8797 and 0.9171. EKF models incorporating skin temperature measurements demonstrated higher sensitivity in most configurations, with values exceeding 0.77 for chest-based and thigh-based observations, while maintaining specificity above 0.85. Phase-dependent EKF variants, including those reported by Rizvi and Falcone, achieved sensitivity values between 0.73 and 0.74, with specificity consistently above 0.90.

Linear Kalman filter models combining heart rate with skin temperature exhibited site-dependent classification performance. Thigh-based configurations achieved sensitivity values close to 0.80, whereas forehead-based configurations showed sensitivity below 0.70, despite maintaining specificity above 0.85. Single-site statistical regression models based solely on skin temperature demonstrated wide variability in sensitivity across anatomical locations, ranging from below 0.30 for upper-back measurements to above 0.55 for abdominal and chest locations, with specificity values generally exceeding 0.75.

On Dataset 2, sensitivity values were lower overall across most methods, while specificity remained comparatively high. Heart-rate–only EKF models yielded sensitivity values between 0.3328 and 0.7180, with specificity values between 0.8324 and 0.8549. EKF models incorporating skin temperature exhibited broader sensitivity ranges, from 0.45 to 0.81, depending on the observation configuration, with specificity values ranging from 0.78 to 0.92. Linear Kalman filter and regression-based models again showed strong site dependence, with some configurations achieving sensitivity above 0.70 while others remained below 0.40.

## 4 Discussion

This study adopted a two-tier analytical approach: first, a systematic review of data-driven methods for core body temperature (CBT) estimation, and second, a standardized benchmark evaluation of the 14 algorithms that were feasible to re-implement using common wearable-derived inputs.

This systematic review of 38 studies provides the most comprehensive synthesis of modeling approaches used for non-invasive estimation of core body temperature during exercise, occupational heat exposure, or environmental stress. The included studies encompass a wide range of participant characteristics, activity protocols, environmental conditions, sensor configurations, and modeling frameworks, resulting in substantial heterogeneity in both methodology and reported performance. The present findings highlight clear trends in the evolution of core-temperature modeling, identify major sources of variability across the literature, and motivate the need for a standardized assessment framework.

Four broad modeling categories were identified across the included studies: Kalman filter or extended Kalman filter (EKF/KF) approaches, statistical models, machine-learning models, and hybrid models integrating physiological models with data-driven components. Statistical models were the most prevalent (17 studies), although EKF/KF approaches were also widely represented (13 studies), particularly in military and occupational settings. Pure machine-learning models were rare, appearing in only three studies, whereas hybrid formulations appeared in five studies.

Across the literature, heart rate was the most common input variable, followed by skin temperature, environmental conditions, load- or activity-related variables, and core temperature history. The diversity of input combinations, including the use of different body temperature sites, insulated skin temperatures, heat-flux measurements, and other specialized variables, was a major contributor to the heterogeneity in reported performance. Similarly, the choice of reference standard varied, with gastrointestinal telemetry pills used in half of the studies and rectal thermometry used in most of the remainder.

Substantial variability was also observed in participant sample size, environmental exposure, and activity protocols. More than half of the studies recruited fewer than 17 participants, and only three included more than 50 participants. Environmental temperatures ranged from cold conditions (<20 ${\;}^{\circ }$C) to hot conditions ($\geq 30{\;}^{\circ }$C), and activity protocols included treadmill walking or running, cycling, load carriage, military patrols, firefighting simulations, and field operations. These factors, combined with uneven reporting of methodological detail, make cross-study comparison difficult and reinforce the need for standardization.

Reported accuracy metrics varied widely across studies. Nevertheless, across the included literature the vast majority of reported RMSE and MAE values fell below the commonly cited 0.5 ${\;}^{\circ }$C threshold for clinical acceptability in core temperature monitoring.

The performance of Kalman filter and EKF models depends heavily on the observation model (e.g., quadratic HR–core temperature mapping, cubic skin temperature formulations), the presence of phase-specific measurement functions, and the environmental or activity context under which the filter is evaluated.

Statistical models demonstrated the greatest structural diversity, ranging from simple single-site regressions to high-dimensional multivariate formulations incorporating skin temperature, heat flux, and environmental variables. The wide variability in their reported performance reflects differences in sensor placement, the number of included sites, and the complexity of the underlying thermal conditions. Moreover, several studies did not report explicit accuracy metrics for these models, further limiting comparability across the literature.

Machine learning approaches, though less common, exhibited a marked dependence on the physiological richness of their input features. Models integrating multimodal or specialized variables such as sweat-related biomarkers or multi-site skin temperature measurements tended to perform substantially better than those relying solely on a single-site skin temperature. Overall, these observations suggest that richer physiological inputs can substantially enhance model fidelity, although many of these signals may be impractical to obtain in typical field settings.

Hybrid models, which combine first-principles thermophysiological formulations with data-driven components, show promise for capturing physiological dynamics while retaining flexibility. However, these approaches also vary widely in structure and parameterization, and the lack of consistent reporting limits deeper comparison.

Taken together, these findings highlight the need for harmonized input definitions, standardized reference measurements, and unified accuracy metrics to enable meaningful cross-study comparisons. Future research would benefit from shared benchmark datasets, transparent reporting of model structures and parameters, and evaluation protocols that reflect realistic deployment conditions.

The review also highlights several broader implications for model development and evaluation. A central challenge is the absence of a common reference framework for comparing methods across studies. The heterogeneity in environmental conditions, sensor configurations, and experimental protocols makes direct comparison difficult, and this issue is further compounded by inconsistent reporting of performance metrics. Several studies provide only partial accuracy measures (e.g., RMSE without MAE or Bland–Altman statistics), while several studies omit numerical performance values entirely. These limitations hinder a clear assessment of relative model performance. This is particularly evident in the case of the widely used Buller 2013 extended Kalman filter: multiple studies have implemented the same method, yet reported different accuracy performances depending on the setting, population, heat exposure, and input availability. When such variability arises for the same method, cross-study comparison becomes even more difficult for distinct algorithms evaluated under heterogeneous protocols. This underscores the rationale for a standardized benchmark framework in which candidate models are re-implemented and evaluated under identical data, input, and evaluation settings.

The input requirements of many published models further constrain their practical deployability and the feasibility of head-to-head comparison. Of the 38 studies included in the systematic review, only 14 could be incorporated into the standardized benchmark analysis because the remaining methods depended on inputs that were not available in the benchmark datasets, such as insulated skin temperatures, multi-site heat-flux measurements, microclimate temperatures, or sweat-derived biomarkers. While these specialized signals can improve model fidelity in tightly controlled laboratory settings, they are often difficult, expensive, or impractical to obtain in real-world environments. In contrast, methods that rely on widely available signals such as heart rate or a single skin-temperature site are naturally aligned with commercially available wearables and therefore have clear translational potential. In the included studies, the heart-rate–only EKF was adopted and evaluated in several independent investigations. Its repeated use reflects not only its methodological simplicity but, more importantly, its practical significance: by relying solely on heart rate, the method remains feasible in real-world athletic, occupational, and field settings where additional sensors may be unavailable, unreliable, or impractical [86], [87], [88]. The popularity of this minimalist-input formulation thus underscores its relevance for scenarios in which low-burden and broadly deployable solutions are essential.

The review also highlights limitations in current data resources for core-temperature modeling. More than half of the included studies enrolled fewer than 20 participants, indicating that many algorithms have been developed and tested on relatively small cohorts. Although such studies can still yield valuable physiological insight, they increase the risk of overfitting and limit the generalizability of reported findings across populations, environments, and clothing or equipment conditions. Together with the restricted input compatibility described above, these observations emphasize the need for larger, more diverse, and better documented datasets, as well as robust data-sharing mechanisms to support independent validation and comparative evaluation.

The standardized benchmark enables direct comparison of reported models under identical inputs, preprocessing, and evaluation settings, and it highlights several systematic performance gaps between the original publications and the reproduced results. For the HR-only EKF method derived from Buller et al. [49], the benchmark RMSE increased from the commonly reported range of ∼0.21–0.41 ${\;}^{\circ }$C across prior studies [49], [50], [51], [77], [78], [79] to $0.4108{\;}^{\circ }\text{C}$ on Dataset 1 and $0.6563{\;}^{\circ }\text{C}$ on Dataset 2 (Table 4). Similar inflation was also observed for phase-dependent EKF variants (Rizvi [46]: reported $0.16{\;}^{\circ }\text{C}$ vs. benchmark $0.3952/0.5400{\;}^{\circ }\text{C}$; Falcone [81]: reported $0.28\pm 0.12{\;}^{\circ }\text{C}$ vs. benchmark $0.3662/0.4921{\;}^{\circ }\text{C}$ for Dataset 1/Dataset 2). These gaps suggest that the performance reported in the literature largely reflects the specific conditions of the study (participant group, environment, activity protocol, and sensor characteristics), rather than just the model structure itself. In particular, most EKF formulations rely on the stability of the HR–CBT mapping; shifts in exercise modality, thermal exposure, or wearable HR noise can directly alter observation residual statistics and thus degrade filtering performance when applied to a different dataset.

Across models, a consistent dataset dependence was observed. For multiple EKF/KF methods and statistical methods, RMSE was markedly higher on Dataset 2 than on Dataset 1. This result suggests that Dataset 2 presents a more challenging estimation setting, potentially due to differences in participant characteristics and activity protocols.

The benchmark also clarifies the effect of incorporating skin-temperature observations. Compared with the HR-only EKF baseline, the Seng EKF (HR + chest skin temperature) achieved lower RMSE on Dataset 1 ($0.3269{\;}^{\circ }\text{C}$ vs. $0.4108{\;}^{\circ }\text{C}$) and improved high-risk detection sensitivity on Dataset 2 (0.8127), demonstrating that direct thermal observations can improve both continuous tracking and threshold-based detection when the selected site provides informative coupling to core temperature. However, the Welles linear KF showed strong site dependence: the variant with thigh and HR outperformed the variant with forehead and HR in continuous tracking on Dataset 1 (0.3618 vs. $0.4489{\;}^{\circ }\text{C}$), whereas on Dataset 2 the results are in contrast (0.6799 vs. $0.5348{\;}^{\circ }\text{C}$). This indicates that the effectiveness of skin sites is not the same across all datasets and may be affected by multiple factors such as different participant groups, environments, activity protocols, and sensor characteristics, leading to dataset-dependent performance.

For high-risk CBT detection, Fig. 6 shows that specificity was generally maintained at a high level across methods, whereas sensitivity exhibited substantially greater variability and was often lower on Dataset 2. Because models were trained and evaluated independently on each dataset, this asymmetry reflects differences in dataset-specific characteristics rather than transfer effects. In particular, sensitivity degradation on Dataset 2 suggests that, within this dataset, model prediction errors during high-temperature periods were more likely to remain below the fixed detection threshold, thereby reducing true-positive detections while preserving true-negative performance. This effect is further illustrated by several configurations that yielded sensitivity values of 0 on Dataset 2 (Table 4), indicating that predicted CBT trajectories rarely exceeded the 38.0 ${\;}^{\circ }$C threshold despite reference measurements doing so. These results illustrate that threshold-based detection outcomes can be highly sensitive to small biases in continuous CBT estimates. In particular, modest underestimation near the 38.0 ${\;}^{\circ }$C threshold can lead to a marked reduction in sensitivity, even when overall continuous error metrics remain moderate. Given this vulnerability to minor threshold fluctuations, the sensitivity value alone appears to be of limited significance as a primary descriptor of model performance. Therefore, we recommend focusing primarily on continuous quality indicators, such as RMSE, which provide a more robust and comprehensive assessment of a model’s tracking fidelity. In this context, sensitivity and specificity are more appropriately utilized as secondary metrics to further investigate and differentiate models that already demonstrate similar RMSE values.

Although the standardized benchmark does not support a single universally best-performing method, several consistent patterns emerge. Across both datasets, EKF-based approaches relying solely on heart rate generally provided stable and interpretable performance and served as a strong baseline, particularly when input availability was limited. Variants that incorporated additional structure, such as phase-dependent observation models or auxiliary skin-temperature measurements, achieved improved accuracy under certain conditions, but these gains were not consistent across datasets.

Methods that depended on specific skin-temperature sites or more complex input configurations exhibited greater variability, with performance strongly influenced by sensor placement, participant characteristics, and experimental context. Taken together, these findings suggest that no single modeling strategy dominates across all settings; rather, simpler heart-rate–based filters are better suited to low-burden, broadly deployable scenarios, whereas multimodal approaches may offer advantages in controlled settings where informative skin-temperature measurements can be reliably obtained [86], [87], [88].

The present work has several limitations. First, although the systematic review has identified 38 eligible studies, only 14 algorithms have been included in the benchmark analysis. This restriction reflects the requirement that all benchmarked models must be reproducible using a standardized set of non-invasive inputs. As a result, several otherwise promising methods that rely on heat-flux measurements, insulated skin temperatures, or other specialized variables have not been eligible for inclusion. Second, model performance has been summarized primarily using aggregate error metrics such as overall RMSE and MAE across all samples. This approach has ensured a consistent population level comparison but may obscure meaningful inter-individual differences that are critical for personalized applications. Participant level metrics have not been emphasized because trial durations have varied considerably between individuals, and simple averaging of participant wise RMSE would have risked giving disproportionate weight to shorter recordings. Third, the benchmark datasets themselves remain relatively small in terms of participant numbers and total trial count. This limits the diversity of physiological responses and environmental exposures represented in the analysis and reduces the statistical power available to evaluate algorithmic robustness across different subgroups or conditions [89]. In addition both datasets have been collected under controlled laboratory conditions and have involved predominantly young, healthy adults. The comparative findings therefore require cautious interpretation when extrapolated to older populations, clinical cohorts, or field-based occupational environments, where thermoregulatory dynamics, wearable sensor performance, and environmental constraints may differ substantially from those captured in the present data.

Recent developments in wearable sensing ecosystems further support the translational potential of non-invasive core temperature estimation. Multi-channel devices integrating inertial measurement units, ECG or PPG-based heart-rate sensors, and increasingly dense skin-temperature arrays are now commonplace in elite sports, military training centres, and occupational monitoring platforms [86], [87], [88], [90]. The growing availability of these multi-sensor systems provides a practical hardware foundation on which more sophisticated core temperature estimation algorithms can be deployed, enabling continuous, high-resolution monitoring under both controlled and free-living conditions.

These technological trends open up a wide range of future application scenarios for core temperature estimation. Accurate, real-time core-temperature monitoring could enable automated cooling or ventilation control systems, real-time heat-risk alerts, and personalized hydration or pacing strategies tailored to an individual’s thermophysiological state [10], [11], [12]. Such applications highlight the broader importance of reliable core temperature estimation, not only as a research problem but as a key enabler for safety, performance optimization, and heat-illness prevention across occupational, military, and athletic domains.

In this systematic review, purely mechanistic or physics-based thermophysiological frameworks, particularly the Predicted Heat Strain (PHS) model [24], were not included in the main systematic search and benchmark evaluation in this study. Because these models primarily rely on predefined parameters rather than continuous, real-time physiological sensor inputs, they fall outside the data-driven and wearable-focused scope defined by the PRISMA eligibility criteria of this study. However, although the present review primarily focuses on data-driven approaches based on wearable physiological sensing, the Predicted Heat Strain (PHS) framework remains a foundational reference model in occupational thermal safety. Therefore, a structured summary of representative developments of this framework is necessary to provide a comprehensive perspective on CBT estimation.

The PHS framework is based on the heat balance of the human body to predict sweat rate and the core/rectal temperature, primarily using environmental parameters (e.g., air temperature, humidity, air velocity, and radiant temperature) together with metabolic rate and clothing insulation. Over the past two decades, several studies have evaluated, validated, and extended the original PHS model to improve its accuracy across occupational, athletic, and individualized monitoring scenarios. Table 5 summarizes representative studies of the PHS framework and its variants.

**Table 5:** Representative studies evaluating and extending Predicted Heat Strain (PHS) framework.

Study	Model details	Input variables	Reference CBT method	Reported metrics
Malchaire [91]	Baseline PHS thermophysiological heat balance model (ISO 7933 revision) using recursive skin-core heat storage partition	Air temperature, humidity, radiation, air velocity, metabolic rate, clothing insulation, body mass, initial rectal temperature	Rectal temperature	Pearson Coefficient: r=0.66 (lab), 0.59 (field), MD: $=0.01\pm 0.39{\;}^{\circ }$C(lab), $=0.01\pm 0.36{\;}^{\circ }$C(field)
Yao [92]	PHS model extended with real-time HR-based metabolic rate estimation for dynamic prediction of in changing activity conditions	Air temperature, humidity, radiation, air velocity, heart rate, clothing insulation, initial skin temperature, exposure time	Mean skin temperature	Mean skin temperature MD:$=0.3{\;}^{\circ }$C, Mean skin temperature SD:$=0.76{\;}^{\circ }$C
Du [25]	Population-specific parameter modification of the baseline PHS model with adjusted initial rectal temperature and HR-based metabolic-rate correction for improved prediction accuracy in Chinese workers	Air temperature, humidity, radiation, air velocity, metabolic rate (estimated from heart rate), clothing insulation, body mass, age, initial rectal temperature	Rectal temperature	$R^{2}$: 0.67 (baseline), Protective efficacy:21.4% (baseline), 71.2%(modified)
Lazaro [93]	PHS model with modified core-temperature prediction equation based on regression between skin temperature and body heat storage	Air temperature, humidity, radiation, air velocity, metabolic rate, clothing insulation, body mass, acclimatization status, posture, skin temperature	Gastrointestinal temperature	Pearson Coefficient: r=0.45, MD: $=-0.023{\;}^{\circ }$C, limits of agreement −1.11 to $0.74{\;}^{\circ }$C
Wang [94]	Individualized PHS model incorporating anthropometric and demographic factors (height, weight, age, and gender) to improve prediction of physiological responses under different environmental conditions	Air temperature, humidity, radiation, air velocity, metabolic rate, clothing insulation, body mass, height, age, gender	Rectal temperature	Maximum difference: $0.74{\;}^{\circ }$C(male,PHS), $0.98{\;}^{\circ }$C(female,PHS), $0.55{\;}^{\circ }$C(male,modified), $0.38{\;}^{\circ }$C (female,modified), RMSE: $0.24{\;}^{\circ }$C(male,modified), $0.20{\;}^{\circ }$C (female,modified),

Overall, subsequent developments of the PHS framework have mainly focused on improving prediction accuracy under dynamic conditions and reducing inter-individual variability. One major development direction is the incorporation of additional anthropometric and demographic characteristics, such as height, age, and sex, into the model structure in order to reduce prediction bias associated with individual variability. These individualized modifications improve the robustness of the PHS framework across different populations and environmental conditions. Another important direction is the integration of wearable physiological measurements, such as heart rate, to replace predefined metabolic-rate parameters and enhance the adaptability of the conventional PHS model to dynamic environmental and activity conditions.

It should also be noted that the PHS model is not designed solely for predicting core body temperature. Instead, it provides predictions of sweat rate and maximum allowable exposure time under heat stress conditions. Consequently, performance metrics reported in the literature for core temperature prediction are not always directly comparable across studies, as different works may report different physiological outputs or criteria. Establishing more standardized evaluation strategies for the PHS framework therefore remains an important topic for future research.

It is important to distinguish between the objectives of PHS models and data-driven wearable-based approaches for core body temperature monitoring. The PHS framework is primarily designed as a ’prediction’ tool for heat strain under predefined environmental and workload scenarios, and is therefore particularly suitable for risk assessment. In contrast, wearable-based methods are typically developed for real-time ’estimation’ of core body temperature, with rapid response to dynamic physiological states and environmental conditions. These two modeling paradigms are therefore complementary. Future research should therefore explore integrated frameworks that combine the predictive capability of PHS models with the real-time estimation capacity of wearable sensing systems, especially for applications such as heat-risk early warning and occupational guidelines.

## CRediT authorship contribution statement

**Yuanzhe Zhao:** Writing – original draft, Visualization, Validation, Software, Resources, Project administration, Methodology, Investigation, Formal analysis, Data curation, Conceptualization. **Weihao Li:** Writing – review & editing, Validation, Methodology, Investigation, Formal analysis, Conceptualization. **Jeroen HM Bergmann:** Writing – review & editing, Supervision, Project administration, Methodology, Investigation, Funding acquisition.

## Declaration of generative AI and AI-assisted technologies in the writing process

During the preparation of this work the author(s) used ChatGPT 5 to refine sentence structure and enhance readability. After using this tool/service, the author(s) reviewed and edited the content as needed and take(s) full responsibility for the content of the published article.

## Funding

This work was supported by the 10.13039/501100000268Biotechnology and Biological Sciences Research Council (BBSRC), grant number UKRI012, and by the National Institute for Health and Care Research (NIHR) HealthTech Research Centre for Community Healthcare at Oxford Health NHS Foundation Trust. The views expressed are those of the author(s) and not necessarily those of the NHS, the NIHR or the Department of Health and Social Care.

## Declaration of competing interest

The authors declare that they have no known competing financial interests or personal relationships that could have appeared to influence the work reported in this paper.

## Data Availability

Data will be made available on request.
